# Supplementary material for: Trait-based predation suitability offers insight into effects of changing prey communities
Source: PeerJ. 2018 Nov 6;6:e5899. doi: 10.7717/peerj.5899 (PMC6225838; doi:10.7717/peerj.5899)
Supplement: Supplemental Information 1 [file peerj-06-5899-s001.docx]

**Table S1:** List of included feeding link references

| **Benthic feeding fish** | **example of feeding reference** |
| --- | --- |
| *Abramis bjoerkna* | 3 |
| *Abramis brama* | 4, 13 |
| *Clupea harengus* | 3, 13, 14 |
| *Coregonus lavaretus* | 12, 3, 7 |
| *Gymnocephalus cernuus* | 9, 10, 13 |
| *Leuciscus idus* | 2 |
| *Osmerus eperlanus* | 3, 11 |
| *Perca fluviatilis* | 8, 13 |
| *Platichthys flesus* | 1, 4, 14 |
| *Rutilus rutilus* | 2, 6, 13 |
| *Triglopsis quadricornis* | 5 |

1. Aarnio, K., Bonsdorff, E. & Rosenback, N. (1996) Food and feeding habits of juvenile flounder platichthys flesus (l.), and turbot scophthalmus maximus l. in the Åland archipelago, northern baltic sea. *Journal of Sea Research*, **36**, 311–320.
2. Brabrand, Å. (1985) Food of roach (Rutilus rutilus) and ide (Leusiscus idus): significance of diet shift for interspecific competition in omnivorous fishes. *Oecologia*, **66**, 461–467.
3. Encyclopedia of Life. (2016) www.eol.org
4. Froese, R. & Pauly, D. (2016) Fishbase, www.fishbase.org
5. Hansson, S., Bengtsson, B.-E. & Bengtsson, Å. (1984) Stomach Contents in Baltic Fourhorn Sculpin ( Myoxocephalus quadricornis L .) with Normal and Deformed Spinal Vertebrae. *Marine Pollution Bulletin*, **15**, 375–377.
6. Lappalainen, A., Rask, M., Koponen, H. & Vesala, S. (2001) Relative abundance, diet and growth of perch(Perca fluviatilis) and roach (Rutilus rutilus) at Tvärminne, northern Baltic Sea, in 1975 and 1997: responses to eutrophication. *Boreal Environment Research*, **6**, 107–118.
7. Merrick, G.W., Hershey, a E. & McDonald, M.E. (1992) Salmonid diet and the size, distribution, and density of benthic invertebrates. *Hydrobiologia*, **240**, 225–233.
8. Mustamäki, N., Cederberg, T. & Mattila, J. (2013) Diet, stable isotopes and morphology of Eurasian perch (Perca fluviatilis) in littoral and pelagic habitats in the northern Baltic Proper. *Environmental Biology of Fishes*.
9. Ogle, D., Selgeby, J.H., Newman, R.M. & Henry, M.G. (1995) Diet and Feeding Periodicity of Ruffe in the St. Louis River Estuary, Lake Superior. *Transactions of the American Fisheries Society*, **124**, 356–369.
10. Pilinkovskij, A., Kesminas, V., Bukelskis, E. & Civas, L. (2014) Ruffe (Gymnocephalus cernuus L.) growth and diet in Lake Dusia. *Arch. Pol. Fish.*, **22**, 110–119.
11. Taal, I., Saks, L., Nedolgova, S., Verliin, A., Kesler, M., Jürgens, K., Svirgsden, R., Vetemaa, M. & Saat, T. (2014) Diet composition of smelt Osmerus eperlanus (Linnaeus) in brackish near-shore ecosystem (Eru Bay, Baltic Sea). *Ecology of Freshwater Fish*, **23**, 121–128.
12. Verliin, A., Kotta, J., Orav-Kotta, H., Saks, L. & Vetemaa, M. (2011) Food selection of Coregonus lavaretus in a brackish water ecosystem. *Journal of Fish Biology*, **78**, 540–551.
13. Winkler, H.M. & Debus, L. (1996) Is the polychaete Marenzelleria viridis an important food item for fish? *Proceedings of the 13th Symposium of the Baltic Marine Biologists*, 147–151.
14. Yletyinen, J., Bodin, Ö., Weigel, B., Nordström, M.C., Bonsdorff, E. & Blenckner, T. (2016) Regime shifts in marine communities: a complex systems perspective on food web dynamics. *Proceedings of the Royal Society B: Biological Sciences*, **283**, 20152569.
