# Supplementary material for: Trait-based predation suitability offers insight into effects of changing prey communities
Source: PeerJ. 2018 Nov 6;6:e5899. doi: 10.7717/peerj.5899 (PMC6225838; doi:10.7717/peerj.5899)
Supplement: Supplemental Information 2 — Binary feeding links between fish predators (columns) and zoobenthic prey (rows). [file peerj-06-5899-s002.docx]

Table S2: Binary feeding links derived from references in table S1

|  | Abramis bjoerkna | Abramis brama | Clupea harengus | Coregonus lavaretus | Gymnocephalus cernuus | Leuciscus idus | Osmerus eperlanus | Perca fluviatilis | Platichthys flesus | Rutilus rutilus | Triglopsis quadricornis |
| --- | --- | --- | --- | --- | --- | --- | --- | --- | --- | --- | --- |
| Halicryptus spinulosus | 0 | 0 | 0 | 0 | 0 | 0 | 0 | 0 | 1 | 0 | 0 |
| Valvata sp. | 0 | 0 | 0 | 1 | 0 | 0 | 0 | 0 | 0 | 0 | 0 |
| Macoma balthica | 0 | 0 | 0 | 1 | 0 | 0 | 0 | 1 | 1 | 1 | 0 |
| Ostracoda | 1 | 1 | 0 | 1 | 0 | 0 | 0 | 1 | 1 | 1 | 0 |
| Oligochaeta | 1 | 1 | 0 | 1 | 0 | 0 | 0 | 1 | 1 | 0 | 0 |
| Hediste diversicolor | 0 | 1 | 0 | 1 | 0 | 0 | 1 | 1 | 0 | 0 | 0 |
| Bithynia tentaculata | 0 | 0 | 0 | 0 | 0 | 0 | 0 | 0 | 0 | 0 | 0 |
| Limapontia capitata | 0 | 0 | 0 | 0 | 0 | 1 | 0 | 0 | 0 | 1 | 0 |
| Cerastoderma glaucum | 0 | 0 | 0 | 0 | 0 | 0 | 0 | 1 | 1 | 1 | 0 |
| Mya arenaria | 0 | 0 | 0 | 0 | 0 | 0 | 0 | 1 | 1 | 0 | 0 |
| Jaera sp | 0 | 0 | 0 | 1 | 0 | 0 | 0 | 1 | 1 | 0 | 0 |
| Saduria entomon | 0 | 0 | 0 | 0 | 0 | 0 | 1 | 1 | 0 | 0 | 1 |
| Chironomidae | 1 | 1 | 0 | 1 | 1 | 1 | 1 | 1 | 1 | 1 | 0 |
| Cyanophtalma obscura | 0 | 0 | 0 | 0 | 0 | 0 | 0 | 0 | 1 | 0 | 0 |
| Marenzelleria spp. | 0 | 1 | 1 | 0 | 1 | 0 | 0 | 1 | 1 | 1 | 0 |
| Pygospio sp. | 0 | 0 | 0 | 0 | 0 | 0 | 0 | 0 | 1 | 0 | 0 |
| Harmothoe sarsi | 0 | 0 | 1 | 0 | 0 | 0 | 0 | 0 | 0 | 0 | 0 |
| Hydrobia sp. | 0 | 0 | 0 | 1 | 0 | 1 | 1 | 1 | 1 | 1 | 0 |
| Potamopyrgus antipodarum | 0 | 1 | 0 | 0 | 0 | 1 | 1 | 0 | 1 | 1 | 0 |
| Theodoxus fluvatilis | 0 | 0 | 0 | 1 | 0 | 0 | 0 | 1 | 1 | 1 | 0 |
| Mytilus edulis | 0 | 0 | 0 | 1 | 0 | 0 | 0 | 1 | 1 | 1 | 0 |
| Corophium volutator | 0 | 1 | 0 | 0 | 0 | 0 | 1 | 1 | 1 | 0 | 0 |
| Gammarus sp. | 0 | 0 | 1 | 1 | 1 | 0 | 1 | 1 | 1 | 0 | 0 |
| Monoporeia affinis | 0 | 0 | 1 | 0 | 0 | 0 | 1 | 0 | 0 | 1 | 1 |
