# Supplementary material for: Trait-based predation suitability offers insight into effects of changing prey communities
Source: PeerJ. 2018 Nov 6;6:e5899. doi: 10.7717/peerj.5899 (PMC6225838; doi:10.7717/peerj.5899)
Supplement: Supplemental Information 4 — This file provides the R code, workflow and results for all GLMMs (all fish and species-specific models). [file peerj-06-5899-s004.pdf]

Workflow and results of GLMM in Weigel & Bonsdorff: Trait-based predation suitability offers insight into effects of changing prey communities

```
#Library
library(lme4)
```

```
## Loading required package: Matrix
```

```
library(lmerTest)
```

```
##
## Attaching package: 'lmerTest'

## The following object is masked from 'package:lme4':
##
##     lmer

## The following object is masked from 'package:stats':
##
##     step
```

```
#Tranform predation suitability values
valueT<-log(value+0.01)
#scale to 0 mean and unit variance
v<-scale(valueT)
#include tranformed suitability values "v" in data frame
data = cbind(v,data)
colnames(data)[1] = "v"
head(data)
```

```
##             v X          ID station exposure year      trait
## 1 -0.9449658 1 S_AlBe1973    AlBe        S 1973        1_S
## 2  1.7354695 2 S_AlBe1973    AlBe        S 1973        1_M
## 3 -1.0715515 3 S_AlBe1973    AlBe        S 1973        1_L
## 4  0.3411671 4 S_AlBe1973    AlBe        S 1973 2_No_prot.
## 5 -0.4992692 5 S_AlBe1973    AlBe        S 1973     2_Tube
## 6 -1.0715515 6 S_AlBe1973    AlBe        S 1973   2_Burrow
##           variable       value
## 1 Abramis_bjoerkna 0.002089864
## 2 Abramis_bjoerkna 0.662486938
## 3 Abramis_bjoerkna 0.000000000
## 4 Abramis_bjoerkna 0.073145246
## 5 Abramis_bjoerkna 0.013584117
## 6 Abramis_bjoerkna 0.000000000
```

```
#year as factor for analysis
yearN<-as.factor(year)

#Fish community model including all 11 fish species
m1 <- lmer(v~yearN+exposure+ (1|station) + (1|variable)+ (1|trait), data)
summary(m1)
```

```
## Linear mixed model fit by REML. t-tests use Satterthwaite's method [
```

```
## lmerModLmerTest]
## Formula: v ~ yearN + exposure + (1 | station) + (1 | variable) + (1 |
##     trait)
##    Data: data
##
## REML criterion at convergence: 36144.3
##
## Scaled residuals:
##     Min      1Q  Median      3Q     Max
## -3.3312 -0.6336 -0.0468  0.6602  4.2921
##
## Random effects:
##  Groups   Name        Variance Std.Dev.
##  trait    (Intercept) 0.507825 0.71262
##  station  (Intercept) 0.009406 0.09698
##  variable (Intercept) 0.013139 0.11463
##  Residual             0.489573 0.69969
## Number of obs: 16896, groups:  trait, 24; station, 16; variable, 11
##
## Fixed effects:
##               Estimate Std. Error         df t value Pr(>|t|)
## (Intercept) -3.192e-02  1.539e-01  2.834e+01  -0.207    0.837
## yearN1989   -3.082e-02  1.523e-02  1.684e+04  -2.024    0.043 *
## yearN2000    9.051e-02  1.523e-02  1.684e+04   5.945 2.82e-09 ***
## yearN2013    1.113e-01  1.523e-02  1.684e+04   7.310 2.78e-13 ***
## exposureS   -2.166e-02  4.967e-02  1.400e+01  -0.436    0.669
## ---
## Signif. codes:  0 '***' 0.001 '**' 0.01 '*' 0.05 '.' 0.1 ' ' 1
##
## Correlation of Fixed Effects:
##           (Intr) yN1989 yN2000 yN2013
## yearN1989 -0.049
## yearN2000 -0.049  0.500
## yearN2013 -0.049  0.500  0.500
## exposureS -0.161  0.000  0.000  0.000
```

```r
hist(resid(m1))
```

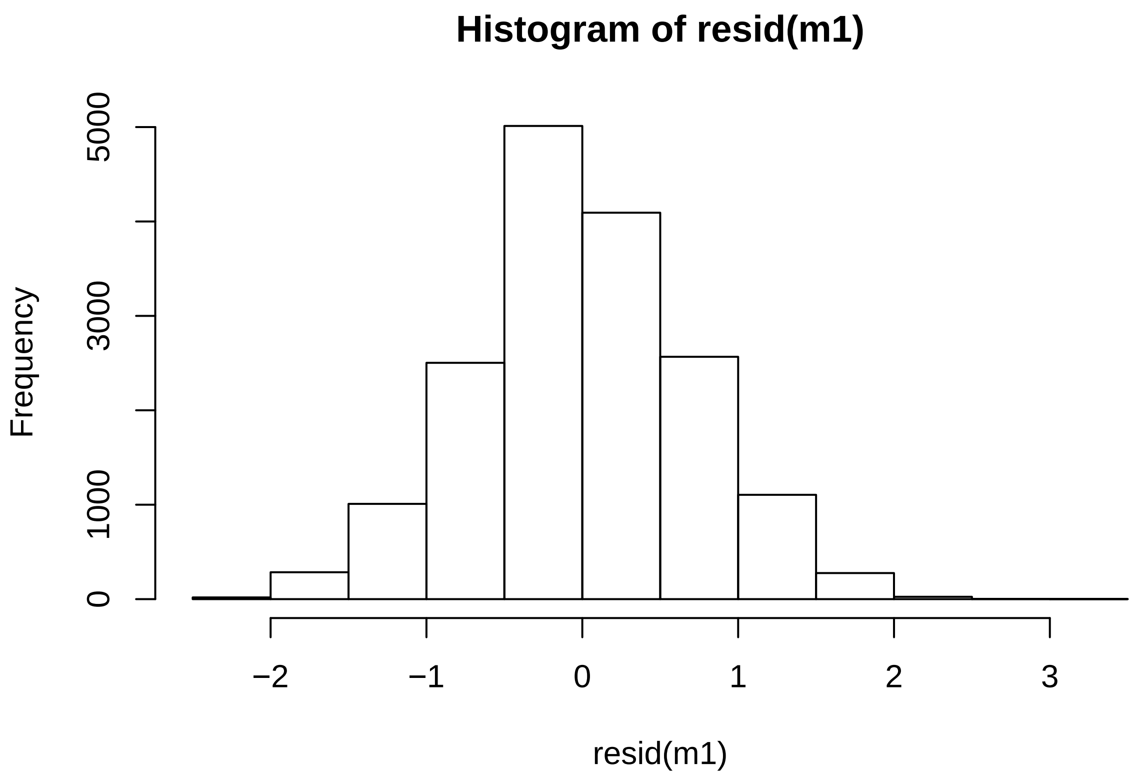


```
#Data for species models
# 1) Define each species data frame from original data frame
Abr_bra <- subset(data, variable=="Abramis_brama")
Abr_bjo <- subset(data, variable=="Abramis_bjoerkna")
Clu<- subset(data, variable=="Clupea_harengus")
Cor <- subset(data, variable=="Coregonus_lavaretus")
Gym<- subset(data, variable=="Gymnocephalus_cernuus")
Leu<- subset(data, variable=="Leuciscus_idus")
Osm<- subset(data, variable=="Osmerus_eperlanus")
Per<- subset(data, variable=="Perca_fluviatilis")
Pla<- subset(data, variable=="Platichthys_flesus")
Rut<- subset(data, variable=="Rutilus_rutilus")
Tri<- subset(data, variable=="Triglopsis_quadricornis")

# 2) Run the models
## MODEL ABRAMIS BRAMA ##
yearABR<-as.factor(Abr_bra$year)
traitABR<-as.factor(Abr_bra$trait)
ABR<-lmer(v~yearABR+exposure+(1|station)+(1|traitABR) ,Abr_bra)
summary(ABR)
```

```
## Linear mixed model fit by REML. t-tests use Satterthwaite's method [
## lmerModLmerTest]
## Formula: v ~ yearABR + exposure + (1 | station) + (1 | traitABR)
##    Data: Abr_bra
##
## REML criterion at convergence: 2984.4
##
## Scaled residuals:
##     Min      1Q  Median      3Q     Max
```

```
## -3.6162 -0.5587  0.0066  0.6681  2.5780
##
## Random effects:
##  Groups   Name        Variance Std.Dev.
##  traitABR (Intercept) 0.580448 0.76187
##  station  (Intercept) 0.007564 0.08697
##  Residual             0.372618 0.61042
## Number of obs: 1536, groups:  traitABR, 24; station, 16
##
## Fixed effects:
##               Estimate Std. Error         df t value Pr(>|t|)
## (Intercept)    0.09607    0.16231   26.59909   0.592  0.55893
## yearABR1989   -0.03913    0.04405 1494.00000  -0.888  0.37460
## yearABR2000    0.09789    0.04405 1494.00000   2.222  0.02643 *
## yearABR2013    0.13031    0.04405 1494.00000   2.958  0.00314 **
## exposureS     -0.03373    0.05349   14.00000  -0.631  0.53848
## ---
## Signif. codes:  0 '***' 0.001 '**' 0.01 '*' 0.05 '.' 0.1 ' ' 1
##
## Correlation of Fixed Effects:
##             (Intr) yABR19 yABR200 yABR201
## yearABR1989 -0.136
## yearABR2000 -0.136  0.500
## yearABR2013 -0.136  0.500  0.500
## exposureS   -0.165  0.000  0.000   0.000
```

```
hist(resid(ABR))
```

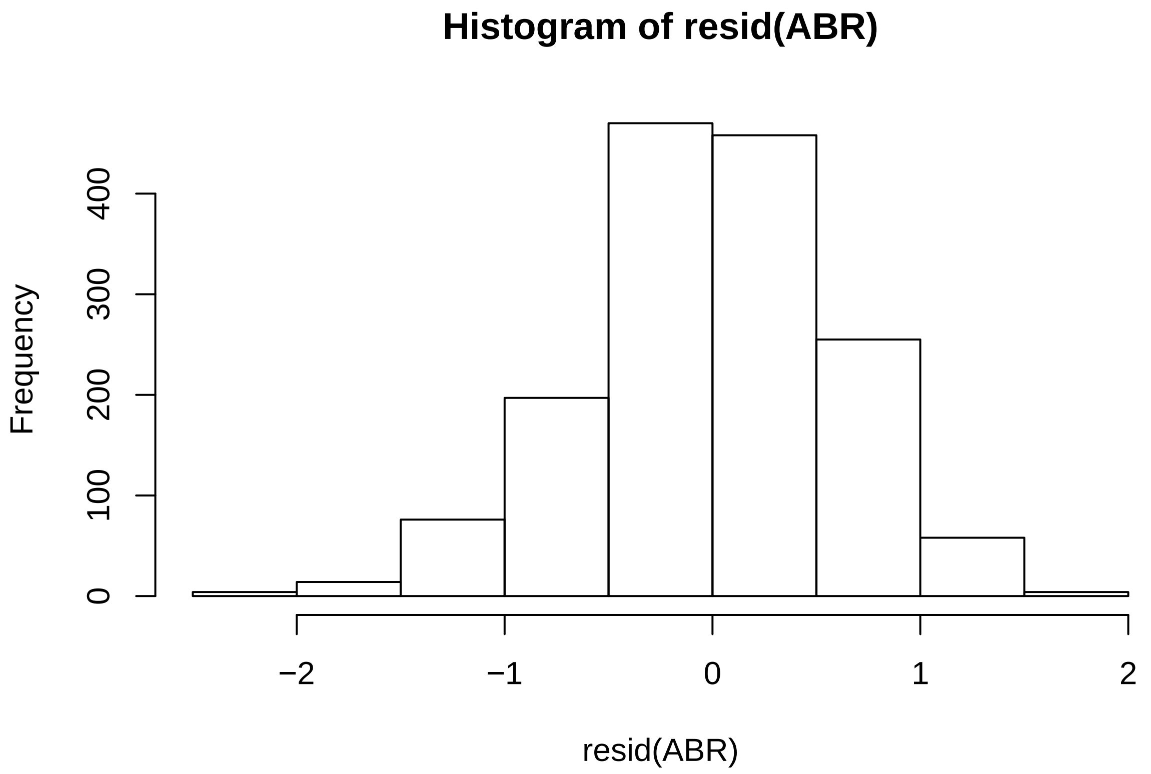


```
## MODEL ABRAMIS BJOERKNA ##
yearABB<-as.factor(Abr_bjo$year)
```

```r
traitABB<-as.factor(Abr_bjo$trait)
ABB<-lmer(v~yearABB+exposure+(1|station)+(1|traitABB) ,Abr_bjo)
summary(ABB)
```

```
## Linear mixed model fit by REML. t-tests use Satterthwaite's method [
## lmerModLmerTest]
## Formula: v ~ yearABB + exposure + (1 | station) + (1 | traitABB)
##    Data: Abr_bjo
##
## REML criterion at convergence: 2795.1
##
## Scaled residuals:
##     Min      1Q  Median      3Q     Max
## -4.2161 -0.3491  0.0402  0.4998  2.6296
##
## Random effects:
##  Groups   Name        Variance Std.Dev.
##  traitABB (Intercept) 0.81832  0.9046
##  station  (Intercept) 0.01142  0.1069
##  Residual             0.32578  0.5708
## Number of obs: 1536, groups:  traitABB, 24; station, 16
##
## Fixed effects:
##                Estimate Std. Error         df t value Pr(>|t|)
## (Intercept)     0.02251    0.19127   26.02781   0.118    0.907
## yearABB1989    -0.02311    0.04119 1494.00000  -0.561    0.575
## yearABB2000    -0.02673    0.04119 1494.00000  -0.649    0.516
## yearABB2013     0.04983    0.04119 1494.00000   1.210    0.227
## exposureS       0.02601    0.06085   14.00000   0.427    0.676
##
## Correlation of Fixed Effects:
##               (Intr) yABB19 yABB200 yABB201
## yearABB1989 -0.108
## yearABB2000 -0.108  0.500
## yearABB2013 -0.108  0.500  0.500
## exposureS   -0.159  0.000  0.000   0.000
```

```r
hist(resid(ABB))
```

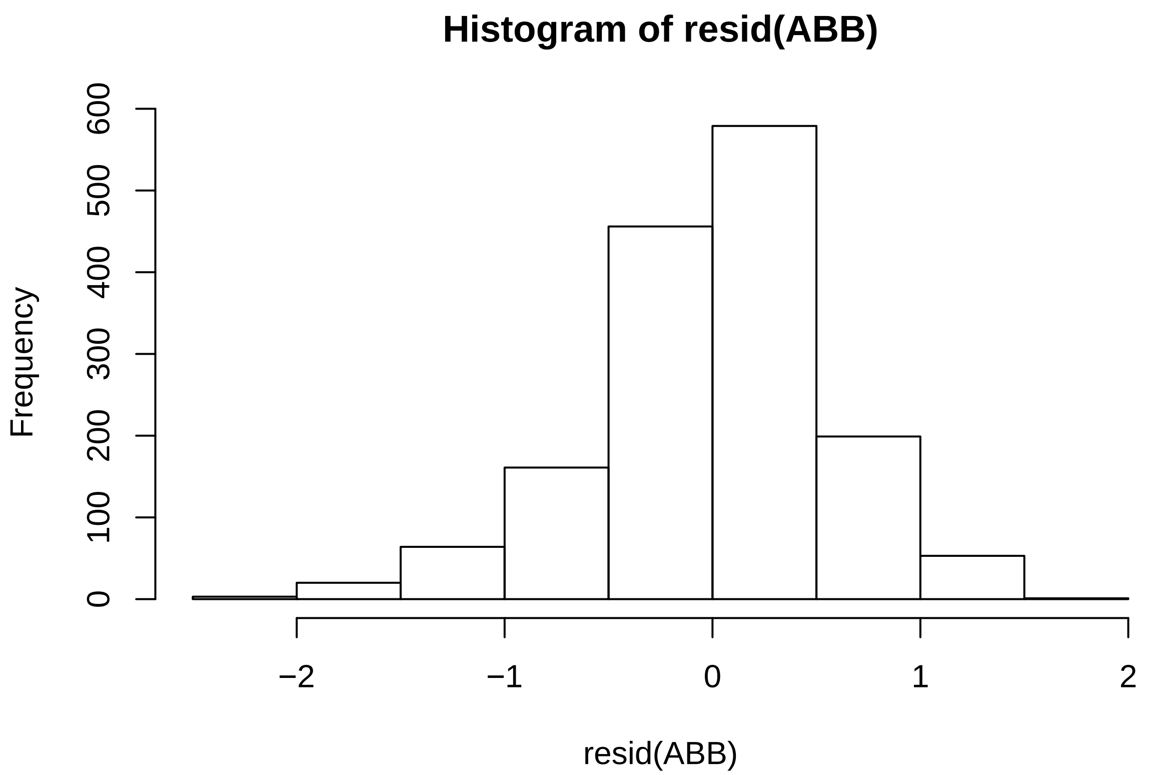


```r
## MODEL CLUPEA HARENGUS ##
yearCLU<-as.factor(Clu$year)
traitCLU<-as.factor(Clu$trait)
CLU<-lmer(v~yearCLU+exposure+(1|station)+(1|traitCLU) ,Clu)
summary(CLU)
```

```
## Linear mixed model fit by REML. t-tests use Satterthwaite's method [
## lmerModLmerTest]
## Formula: v ~ yearCLU + exposure + (1 | station) + (1 | traitCLU)
##    Data: Clu
##
## REML criterion at convergence: 2718
##
## Scaled residuals:
##     Min      1Q  Median      3Q     Max
## -4.3859 -0.3228  0.0335  0.4450  3.9450
##
## Random effects:
##  Groups   Name        Variance Std.Dev.
##  traitCLU (Intercept) 0.706376 0.84046
##  station  (Intercept) 0.002293 0.04788
##  Residual             0.312949 0.55942
## Number of obs: 1536, groups:  traitCLU, 24; station, 16
##
## Fixed effects:
##                  Estimate Std. Error         df t value Pr(>|t|)
## (Intercept)     -0.18771    0.17532   24.71925  -1.071 0.294655
## yearCLU1989     -0.06239    0.04037 1493.99999  -1.545 0.122482
## yearCLU2000      0.14754    0.04037 1493.99999   3.654 0.000267 ***
## yearCLU2013      0.15864    0.04037 1493.99999   3.930  8.9e-05 ***
```

```
## exposureS      -0.03010    0.03726   14.00000  -0.808 0.432636
## ---
## Signif. codes:  0 '***' 0.001 '**' 0.01 '*' 0.05 '.' 0.1 ' ' 1
##
## Correlation of Fixed Effects:
##             (Intr) yCLU19 yCLU200 yCLU201
## yearCLU1989 -0.115
## yearCLU2000 -0.115  0.500
## yearCLU2013 -0.115  0.500  0.500
## exposureS   -0.106  0.000  0.000   0.000
```

```
hist(resid(CLU))
```

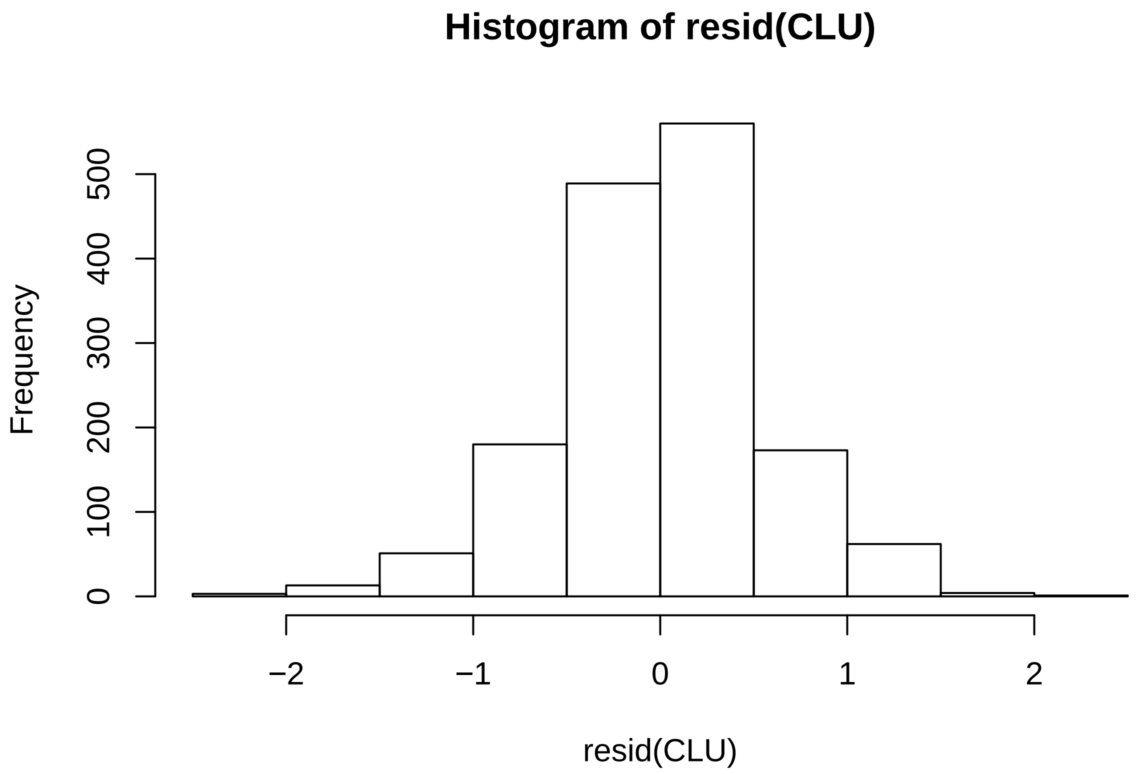


```
## MODEL COREGONUS LAVARETUS ##
yearCOR<-as.factor(Cor$year)
traitCOR<-as.factor(Cor$trait)
COR<-lmer(v~yearCOR+exposure+(1|station)+(1|traitCOR) ,Cor)
summary(COR)
```

```
## Linear mixed model fit by REML. t-tests use Satterthwaite's method [
## lmerModLmerTest]
## Formula: v ~ yearCOR + exposure + (1 | station) + (1 | traitCOR)
##    Data: Cor
##
## REML criterion at convergence: 2821.7
##
## Scaled residuals:
##     Min      1Q  Median      3Q     Max
## -3.7337 -0.5253  0.0050  0.6562  2.8339
##
## Random effects:
```

```
##  Groups   Name        Variance Std.Dev.
##  traitCOR (Intercept) 0.606791 0.77897
##  station  (Intercept) 0.008622 0.09285
##  Residual             0.333777 0.57773
## Number of obs: 1536, groups:  traitCOR, 24; station, 16
##
## Fixed effects:
##                Estimate Std. Error         df t value Pr(>|t|)
## (Intercept)     0.04040    0.16567   26.49290   0.244   0.8092
## yearCOR1989    -0.02068    0.04169 1494.00000  -0.496   0.6199
## yearCOR2000     0.08003    0.04169 1494.00000   1.919   0.0551 .
## yearCOR2013     0.09347    0.04169 1494.00000   2.242   0.0251 *
## exposureS      -0.04579    0.05500   14.00000  -0.833   0.4191
## ---
## Signif. codes:  0 '***' 0.001 '**' 0.01 '*' 0.05 '.' 0.1 ' ' 1
##
## Correlation of Fixed Effects:
##             (Intr) yCOR19 yCOR200 yCOR201
## yearCOR1989 -0.126
## yearCOR2000 -0.126  0.500
## yearCOR2013 -0.126  0.500  0.500
## exposureS   -0.166  0.000  0.000   0.000
```

```
hist(resid(COR))
```

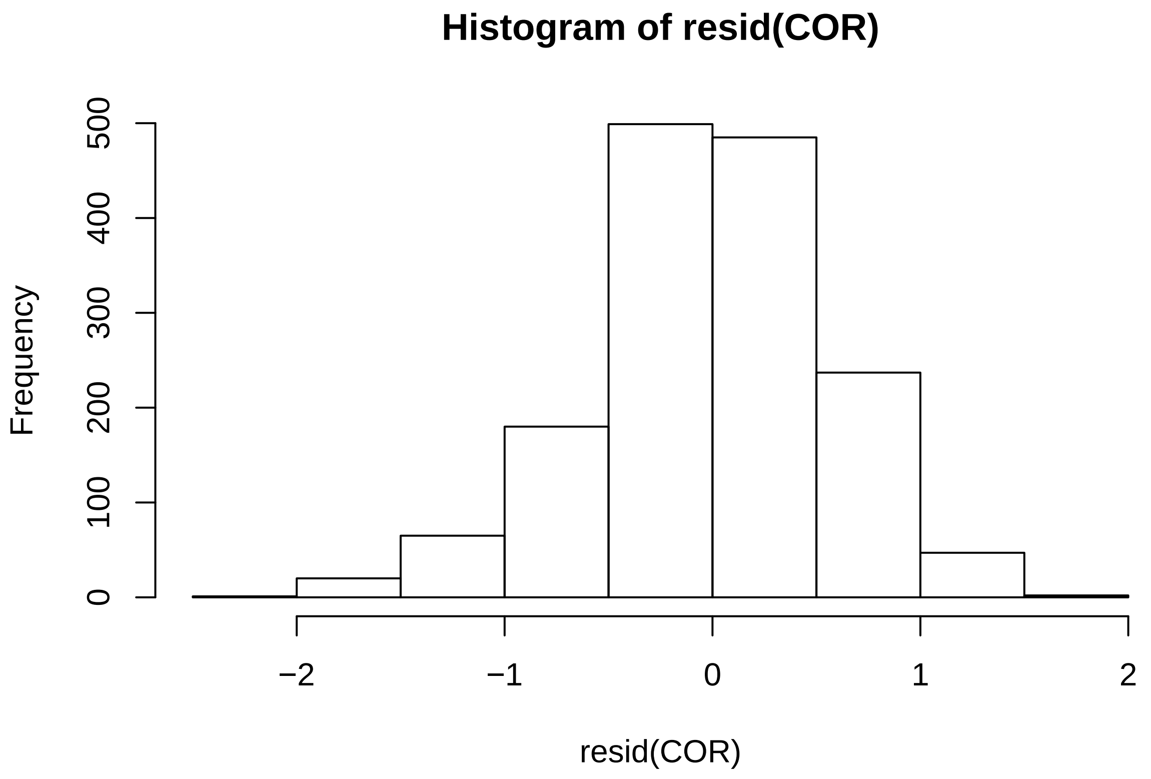


```
## MODEL GYMNOCEPHALUS CERNUUS ##
yearGYM<-as.factor(Gym$year)
traitGYM<-as.factor(Gym$trait)
GYM<-lmer(v~yearGYM+exposure+(1|station)+(1|traitGYM) ,Gym)
summary(GYM)
```

```
## Linear mixed model fit by REML. t-tests use Satterthwaite's method [
## lmerModLmerTest]
## Formula: v ~ yearGYM + exposure + (1 | station) + (1 | traitGYM)
##    Data: Gym
##
## REML criterion at convergence: 2940.1
##
## Scaled residuals:
##     Min      1Q  Median      3Q     Max
## -3.9974 -0.4814  0.0162  0.5369  3.2101
##
## Random effects:
##  Groups   Name        Variance Std.Dev.
##  traitGYM (Intercept) 0.734391 0.85697
##  station  (Intercept) 0.009062 0.09519
##  Residual             0.360056 0.60005
## Number of obs: 1536, groups:  traitGYM, 24; station, 16
##
## Fixed effects:
##                Estimate Std. Error         df t value Pr(>|t|)
## (Intercept)    -0.05406    0.18140   26.07674  -0.298  0.76804
## yearGYM1989    -0.05648    0.04330 1494.00000  -1.304  0.19235
## yearGYM2000     0.12604    0.04330 1494.00000   2.911  0.00366 **
## yearGYM2013     0.17314    0.04330 1494.00000   3.998 6.69e-05 ***
## exposureS       0.02354    0.05660   14.00000   0.416  0.68379
## ---
## Signif. codes:  0 '***' 0.001 '**' 0.01 '*' 0.05 '.' 0.1 ' ' 1
##
## Correlation of Fixed Effects:
##             (Intr) yGYM19 yGYM200 yGYM201
## yearGYM1989 -0.119
## yearGYM2000 -0.119  0.500
## yearGYM2013 -0.119  0.500  0.500
## exposureS   -0.156  0.000  0.000   0.000
```

```r
hist(resid(GYM))
```

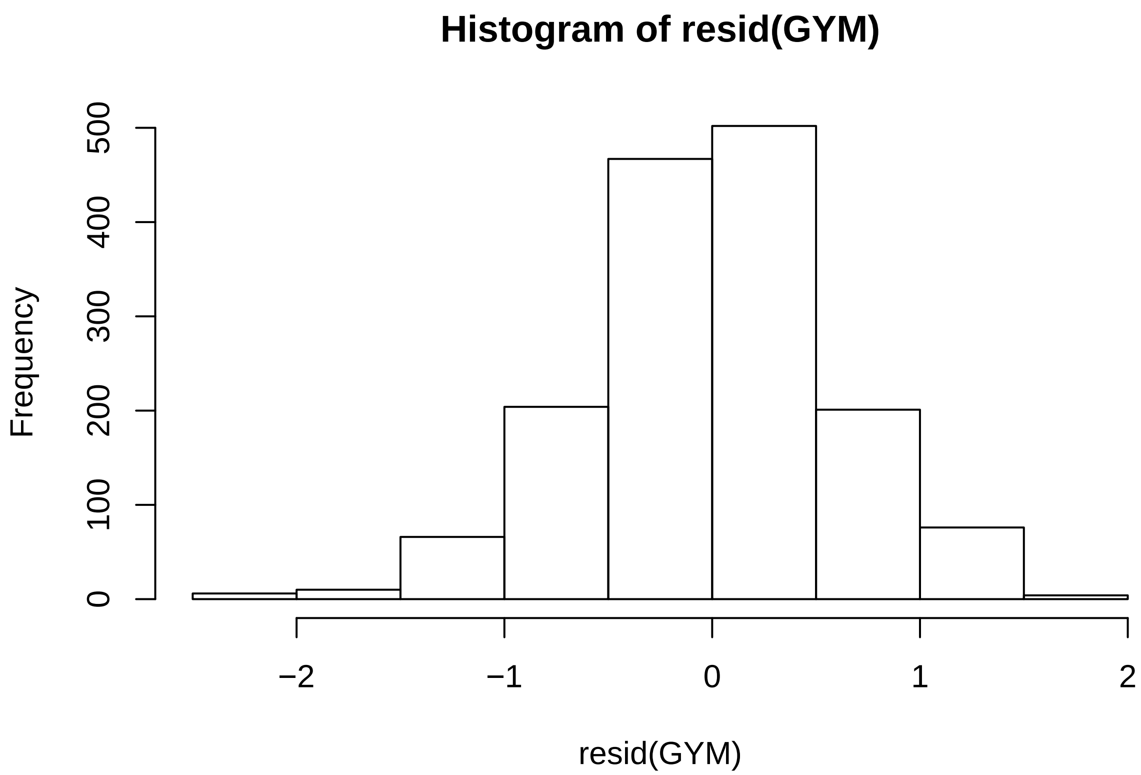


```r
## MODEL LEUCISCUS IDUS ##
yearLEU<-as.factor(Leu$year)
traitLEU<-as.factor(Leu$trait)
LEU<-lmer(v~yearLEU+exposure+(1|station)+(1|traitLEU) ,Leu)
summary(LEU)
```

```
## Linear mixed model fit by REML. t-tests use Satterthwaite's method [
## lmerModLmerTest]
## Formula: v ~ yearLEU + exposure + (1 | station) + (1 | traitLEU)
##    Data: Leu
##
## REML criterion at convergence: 2664.7
##
## Scaled residuals:
##     Min      1Q  Median      3Q     Max
## -4.5224 -0.3360  0.0404  0.4728  2.6488
##
## Random effects:
##  Groups   Name        Variance Std.Dev.
##  traitLEU (Intercept) 0.74363  0.8623
##  station  (Intercept) 0.01157  0.1076
##  Residual             0.29901  0.5468
## Number of obs: 1536, groups:  traitLEU, 24; station, 16
##
## Fixed effects:
##                 Estimate Std. Error         df t value Pr(>|t|)
## (Intercept)     -0.12590    0.18277   26.24970  -0.689   0.4969
## yearLEU1989     -0.01865    0.03946 1494.00001  -0.473   0.6366
## yearLEU2000      0.02952    0.03946 1494.00001   0.748   0.4546
## yearLEU2013      0.07380    0.03946 1494.00001   1.870   0.0617 .
```

```
## exposureS      0.04312    0.06059   14.00000   0.712   0.4883
## ---
## Signif. codes:  0 '***' 0.001 '**' 0.01 '*' 0.05 '.' 0.1 ' ' 1
##
## Correlation of Fixed Effects:
##             (Intr) yLEU19 yLEU200 yLEU201
## yearLEU1989 -0.108
## yearLEU2000 -0.108  0.500
## yearLEU2013 -0.108  0.500  0.500
## exposureS   -0.166  0.000  0.000   0.000
```

```
hist(resid(LEU))
```

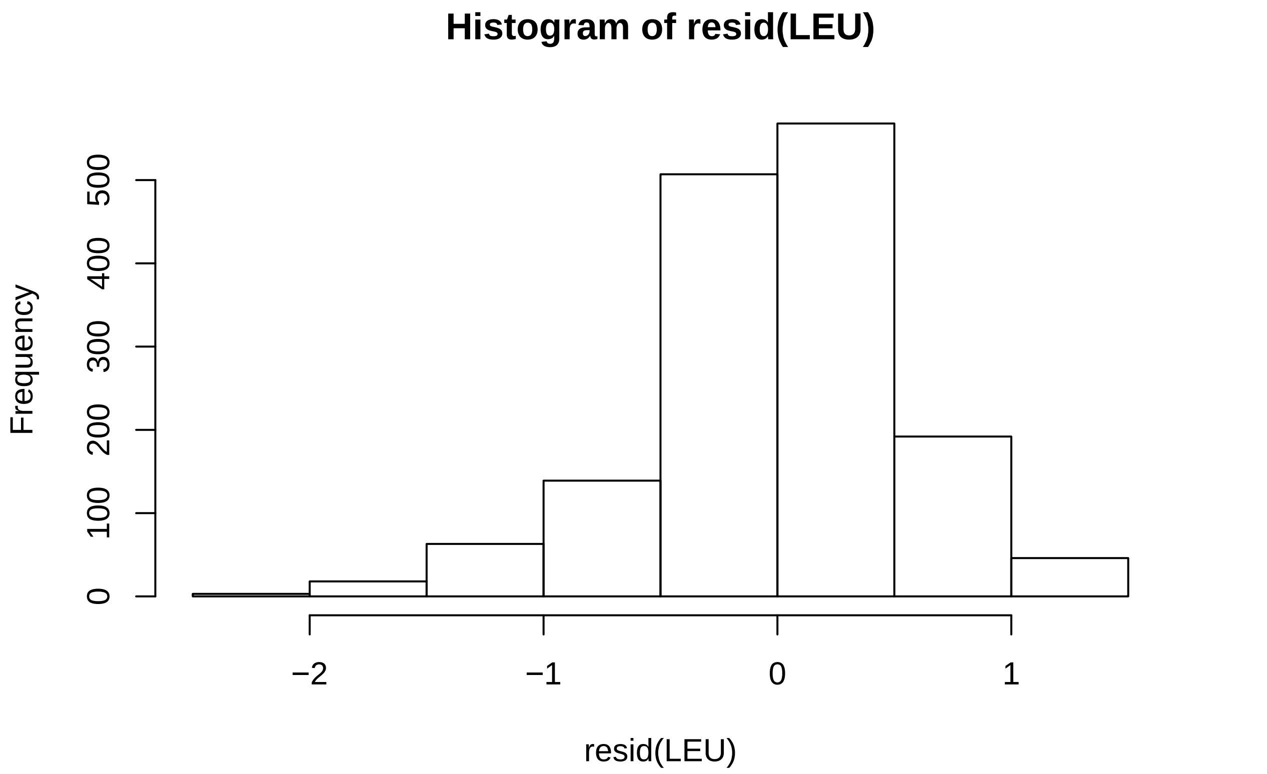


```
## MODEL LEUCISCUS IDUS ##
yearOSM<-as.factor(Osm$year)
traitOSM<-as.factor(Osm$trait)
OSM<-lmer(v~yearOSM+exposure+(1|station)+(1|traitOSM) ,Osm)
summary(OSM)
```

```
## Linear mixed model fit by REML. t-tests use Satterthwaite's method [
## lmerModLmerTest]
## Formula: v ~ yearOSM + exposure + (1 | station) + (1 | traitOSM)
##    Data: Osm
##
## REML criterion at convergence: 2870.5
##
## Scaled residuals:
##     Min      1Q  Median      3Q     Max
## -3.9013 -0.5277  0.0013  0.5953  3.5770
##
## Random effects:
```

```
##  Groups   Name        Variance Std.Dev.
##  traitOSM (Intercept) 0.611795 0.78217
##  station  (Intercept) 0.007585 0.08709
##  Residual             0.345085 0.58744
## Number of obs: 1536, groups:  traitOSM, 24; station, 16
##
## Fixed effects:
##              Estimate Std. Error         df t value Pr(>|t|)
## (Intercept)   -0.01201    0.16602   26.29437  -0.072   0.9429
## yearOSM1989   -0.02308    0.04239 1494.00000  -0.544   0.5862
## yearOSM2000    0.10292    0.04239 1494.00000   2.428   0.0153 *
## yearOSM2013    0.10644    0.04239 1494.00000   2.511   0.0122 *
## exposureS     -0.01704    0.05287   14.00000  -0.322   0.7520
## ---
## Signif. codes:  0 '***' 0.001 '**' 0.01 '*' 0.05 '.' 0.1 ' ' 1
##
## Correlation of Fixed Effects:
##             (Intr) yOSM19 yOSM200 yOSM201
## yearOSM1989 -0.128
## yearOSM2000 -0.128  0.500
## yearOSM2013 -0.128  0.500  0.500
## exposureS   -0.159  0.000  0.000   0.000
```

```
hist(resid(OSM))
```

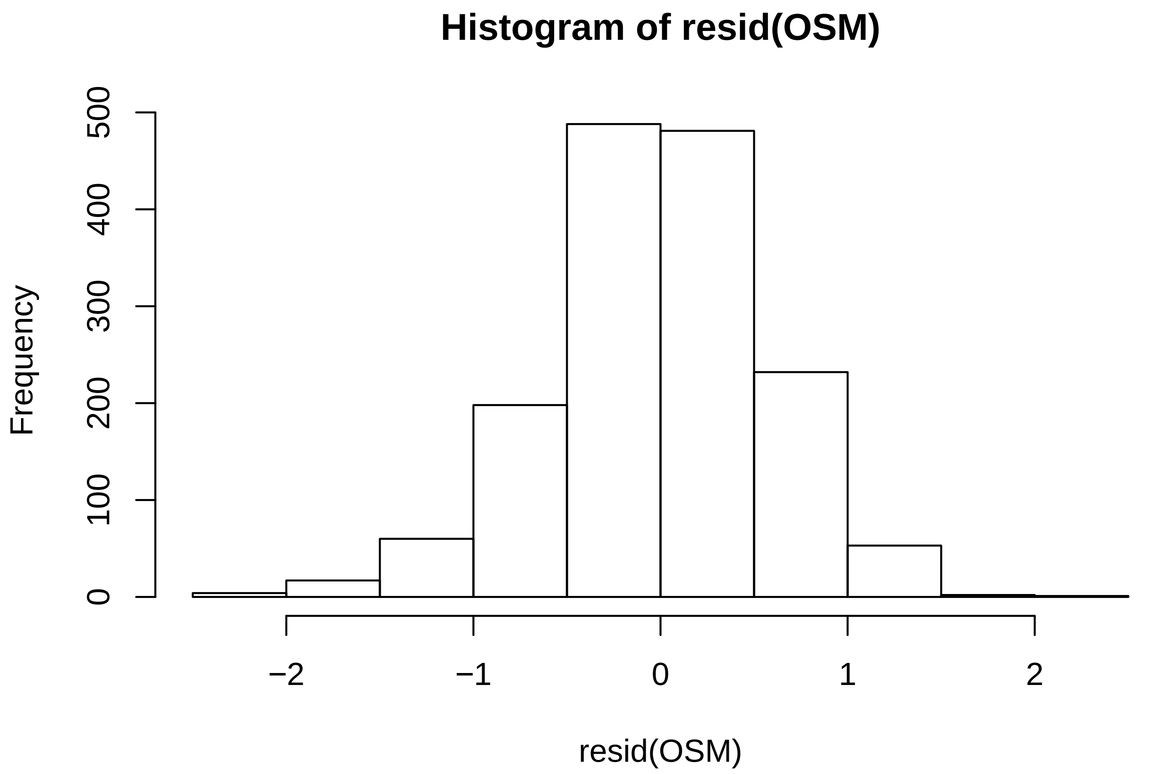


```
## MODEL PERCA FLUVIATILIS ##
yearPER<-as.factor(Per$year)
traitPER<-as.factor(Per$trait)
PER<-lmer(v~yearPER+exposure+(1|station)+(1|traitPER) ,Per)
summary(PER)
```

```
## Linear mixed model fit by REML. t-tests use Satterthwaite's method [
## lmerModLmerTest]
## Formula: v ~ yearPER + exposure + (1 | station) + (1 | traitPER)
##    Data: Per
##
## REML criterion at convergence: 2919.9
##
## Scaled residuals:
##     Min      1Q  Median      3Q     Max
## -3.4150 -0.5583  0.0036  0.6728  3.0005
##
## Random effects:
##  Groups   Name        Variance Std.Dev.
##  traitPER (Intercept) 0.552575 0.74335
##  station  (Intercept) 0.007571 0.08701
##  Residual             0.357193 0.59766
## Number of obs: 1536, groups:  traitPER, 24; station, 16
##
## Fixed effects:
##                Estimate Std. Error         df t value Pr(>|t|)
## (Intercept)     0.05725    0.15853   26.69965   0.361  0.72085
## yearPER1989    -0.02545    0.04313 1494.00000  -0.590  0.55525
## yearPER2000     0.10609    0.04313 1494.00000   2.460  0.01402 *
## yearPER2013     0.12095    0.04313 1494.00000   2.804  0.00511 **
## exposureS      -0.05068    0.05313   14.00000  -0.954  0.35628
## ---
## Signif. codes:  0 '***' 0.001 '**' 0.01 '*' 0.05 '.' 0.1 ' ' 1
##
## Correlation of Fixed Effects:
##             (Intr) yPER19 yPER200 yPER201
## yearPER1989 -0.136
## yearPER2000 -0.136  0.500
## yearPER2013 -0.136  0.500  0.500
## exposureS   -0.168  0.000  0.000   0.000
```

```r
hist(resid(PER))
```

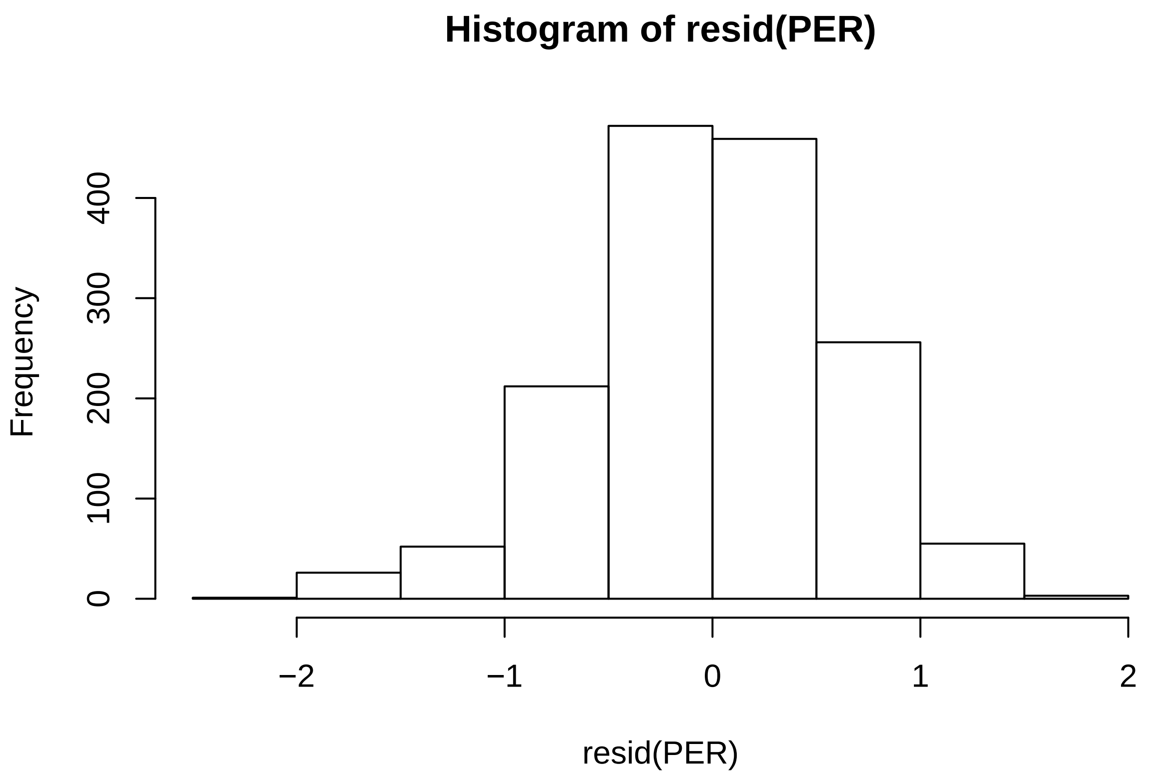


```r
## MODEL PLATICHTHYS FLESUS ##
yearPLA<-as.factor(Pla$year)
traitPLA<-as.factor(Pla$trait)
PLA<-lmer(v~yearPLA+exposure+(1|station)+(1|traitPLA) ,Pla)
summary(PLA)
```

```
## Linear mixed model fit by REML. t-tests use Satterthwaite's method [
## lmerModLmerTest]
## Formula: v ~ yearPLA + exposure + (1 | station) + (1 | traitPLA)
##    Data: Pla
##
## REML criterion at convergence: 2924.3
##
## Scaled residuals:
##     Min      1Q  Median      3Q     Max
## -3.6632 -0.5800  0.0122  0.6821  2.6777
##
## Random effects:
##  Groups   Name        Variance Std.Dev.
##  traitPLA (Intercept) 0.551455 0.74260
##  station  (Intercept) 0.007416 0.08612
##  Residual             0.358299 0.59858
## Number of obs: 1536, groups:  traitPLA, 24; station, 16
##
## Fixed effects:
##                 Estimate Std. Error         df t value Pr(>|t|)
## (Intercept)      0.06410    0.15834   26.67600   0.405  0.68884
## yearPLA1989     -0.03602    0.04320 1494.00000  -0.834  0.40458
## yearPLA2000      0.10348    0.04320 1494.00000   2.395  0.01673 *
## yearPLA2013      0.12563    0.04320 1494.00000   2.908  0.00369 **
```

```
## exposureS     -0.05553    0.05279   14.00000  -1.052  0.31073
## ---
## Signif. codes:  0 '***' 0.001 '**' 0.01 '*' 0.05 '.' 0.1 ' ' 1
##
## Correlation of Fixed Effects:
##             (Intr) yPLA19 yPLA200 yPLA201
## yearPLA1989 -0.136
## yearPLA2000 -0.136  0.500
## yearPLA2013 -0.136  0.500  0.500
## exposureS   -0.167  0.000  0.000   0.000
```

```
hist(resid(PLA))
```

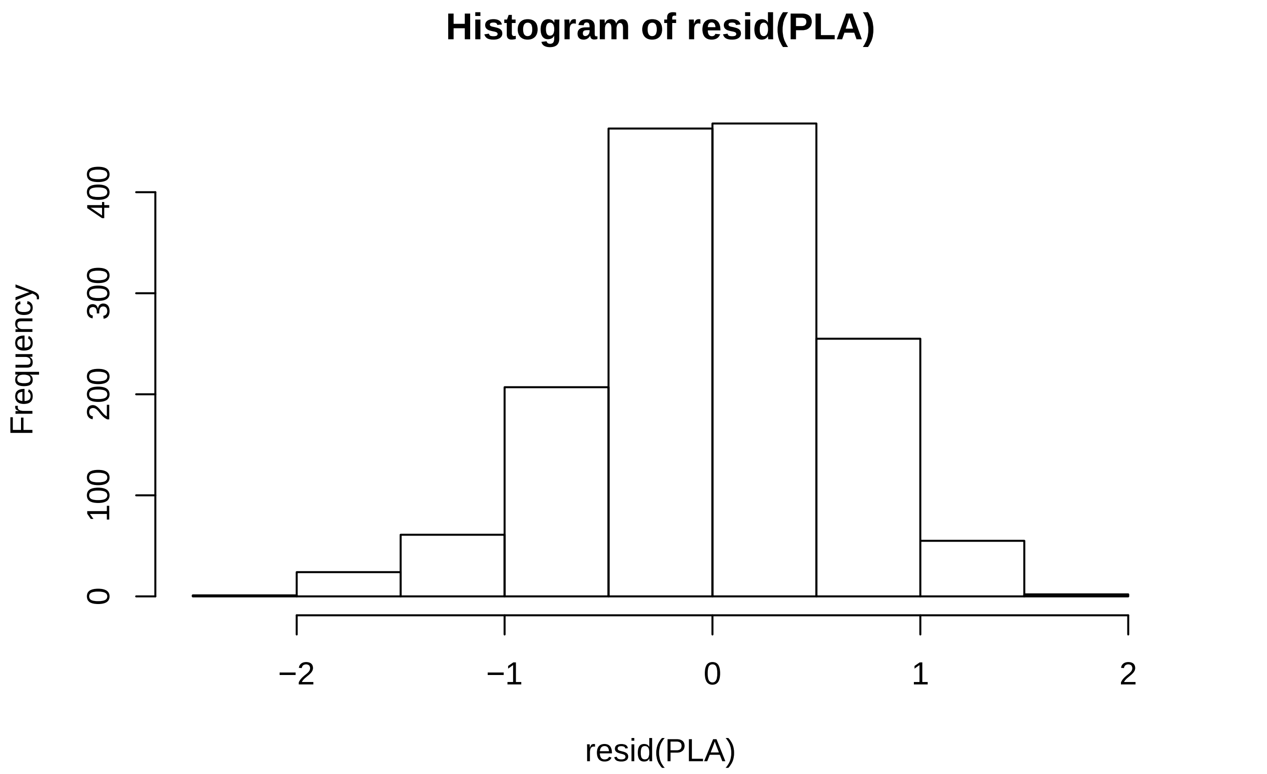


```
## MODEL RUTILUS RUTILUS ##
yearRUT<-as.factor(Rut$year)
traitRUT<-as.factor(Rut$trait)
RUT<-lmer(v~yearRUT+exposure+(1|station)+(1|traitRUT) ,Rut)
summary(RUT)
```

```
## Linear mixed model fit by REML. t-tests use Satterthwaite's method [
## lmerModLmerTest]
## Formula: v ~ yearRUT + exposure + (1 | station) + (1 | traitRUT)
##    Data: Rut
##
## REML criterion at convergence: 2857.1
##
## Scaled residuals:
##     Min      1Q  Median      3Q     Max
## -3.6616 -0.5327  0.0053  0.6593  2.7228
##
## Random effects:
```

```
##  Groups   Name        Variance Std.Dev.
##  traitRUT (Intercept) 0.577903 0.76020
##  station  (Intercept) 0.008163 0.09035
##  Residual             0.342145 0.58493
## Number of obs: 1536, groups:  traitRUT, 24; station, 16
##
## Fixed effects:
##              Estimate Std. Error         df t value Pr(>|t|)
## (Intercept)   0.03934    0.16191   26.60103   0.243  0.80989
## yearRUT1989  -0.02901    0.04221 1494.00000  -0.687  0.49207
## yearRUT2000   0.09586    0.04221 1494.00000   2.271  0.02329 *
## yearRUT2013   0.11648    0.04221 1494.00000   2.759  0.00586 **
## exposureS    -0.05368    0.05415   14.00000  -0.991  0.33830
## ---
## Signif. codes:  0 '***' 0.001 '**' 0.01 '*' 0.05 '.' 0.1 ' ' 1
##
## Correlation of Fixed Effects:
##             (Intr) yRUT19 yRUT200 yRUT201
## yearRUT1989 -0.130
## yearRUT2000 -0.130  0.500
## yearRUT2013 -0.130  0.500  0.500
## exposureS   -0.167  0.000  0.000   0.000
```

```
hist(resid(RUT))
```

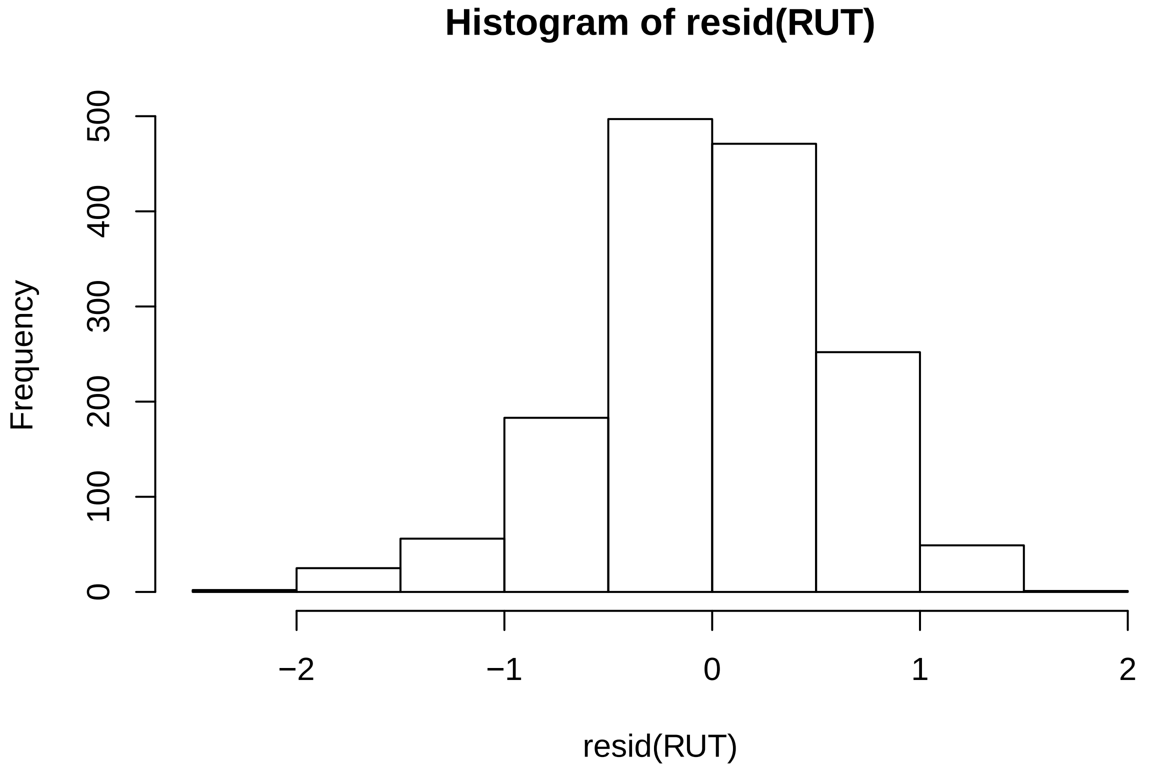


```
## MODEL TRIGLOPSIS QUADRICORNIS ##
yearTRI<-as.factor(Tri$year)
traitTRI<-as.factor(Tri$trait)
TRI<-lmer(v~yearTRI+exposure+exposure+(1|station)+(1|traitTRI),Tri)
summary(TRI)
```

```
## Linear mixed model fit by REML. t-tests use Satterthwaite's method [
## lmerModLmerTest]
## Formula:
## v ~ yearTRI + exposure + exposure + (1 | station) + (1 | traitTRI)
##    Data: Tri
##
## REML criterion at convergence: 2611.4
##
## Scaled residuals:
##     Min      1Q  Median      3Q     Max
## -4.5078 -0.2066  0.0091  0.2613  4.3104
##
## Random effects:
##  Groups   Name        Variance Std.Dev.
##  traitTRI (Intercept) 0.877476 0.93674
##  station  (Intercept) 0.004735 0.06881
##  Residual             0.289530 0.53808
## Number of obs: 1536, groups:  traitTRI, 24; station, 16
##
## Fixed effects:
##               Estimate Std. Error        df t value Pr(>|t|)
## (Intercept) -2.911e-01  1.952e-01  2.469e+01  -1.491 0.148521
## yearTRI1989 -4.973e-03  3.883e-02  1.494e+03  -0.128 0.898106
## yearTRI2000  1.330e-01  3.883e-02  1.494e+03   3.424 0.000633 ***
## yearTRI2013  7.563e-02  3.883e-02  1.494e+03   1.948 0.051646 .
## exposureS   -4.437e-02  4.402e-02  1.400e+01  -1.008 0.330636
## ---
## Signif. codes:  0 '***' 0.001 '**' 0.01 '*' 0.05 '.' 0.1 ' ' 1
##
## Correlation of Fixed Effects:
##             (Intr) yTRI19 yTRI200 yTRI201
## yearTRI1989 -0.099
## yearTRI2000 -0.099  0.500
## yearTRI2013 -0.099  0.500  0.500
## exposureS   -0.113  0.000  0.000   0.000
```

```
hist(resid(TRI))
```

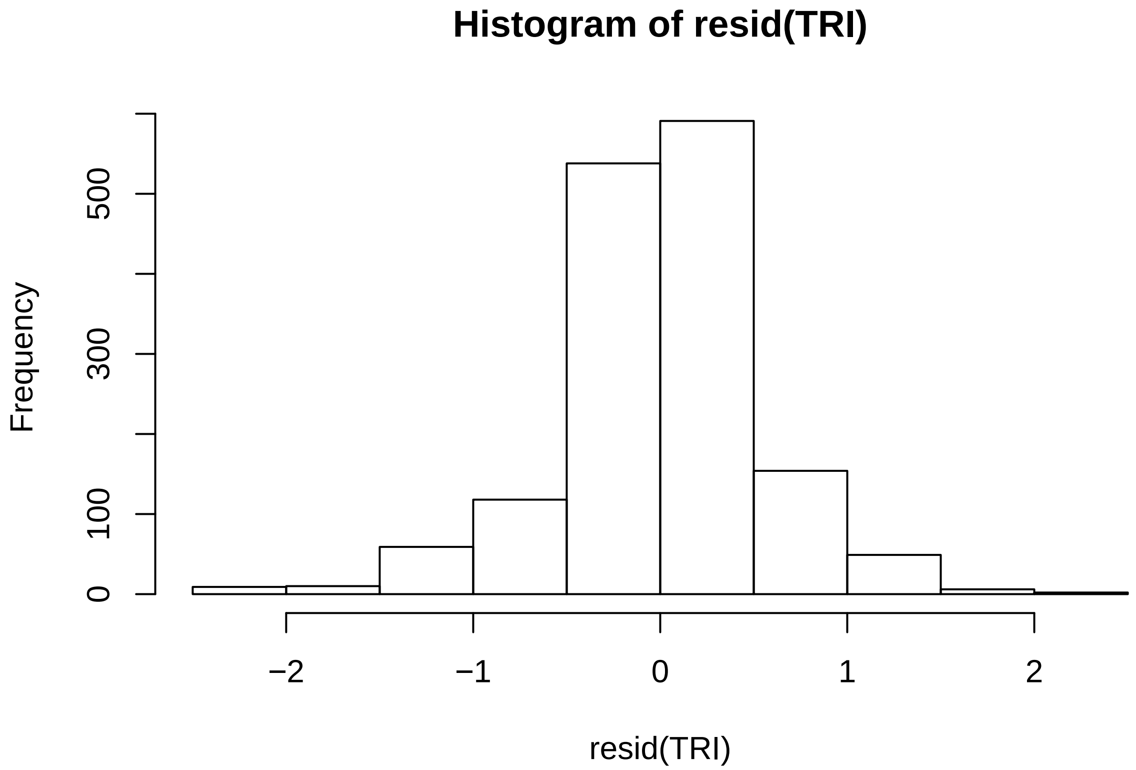
Histogram of resid(TRI)
Frequency
0
100
300
500
−2
−1
0
1
2
resid(TRI)
